# The effect of image quality on the reliability of OCT angiography measurements in patients with diabetes

**DOI:** 10.1186/s40942-019-0197-4

**Published:** 2019-11-04

**Authors:** Cecília Czakó, Lilla István, Mónika Ecsedy, Zsuzsa Récsán, Gábor Sándor, Fruzsina Benyó, Hajnalka Horváth, András Papp, Miklós Resch, Ágnes Borbándy, Zoltán Zsolt Nagy, Illés Kovács

**Affiliations:** Department of Ophthalmology, Semmelweis University, 26 Üllői Street, Budapest, 1085 Hungary

**Keywords:** OCT angiography, Image quality, Retinal vessel density, Diabetic retinopathy

## Abstract

**Background:**

This study aimed to determine the relationship between image quality and measurement repeatability of optical coherence tomography angiography (OCTA) parameters in patients with non-proliferative diabetic retinopathy.

**Methods:**

A total of 100 eyes of 50 patients were included in the study. Three OCTA images were obtained consecutively during one session of imaging in all patients using the RTVue AngioVue OCTA device. We applied the signal strength index (SSI) provided by the RTVue system to define scan quality. Superficial vessel density (VD) in the central 3 × 3 mm macular and in the perifoveal region, as well as foveal avascular zone (FAZ) area were evaluated by the AngioAnalytics software for each scan from three consecutive measurements, whereby measurement repeatability of the OCTA parameters were calculated. The effect of SSI value on OCTA parameters, as well as on measurement errors was assessed.

**Results:**

Values of SSI ranged from 30 to 85 with an overall mean of 61.79 ± 10.38. Mean SSI values showed significant positive correlation with the mean retinal capillary vessel density values, but not with non-flow area. Repeatability of OCTA parameters was generally improved with higher SSI values. We calculated a mean correction factor of 0.22% (95% CI 0.20–0.24 µm; p < 0.001) for VD at the 3 × 3 mm macular scan, 0.23% (95% CI 0.21–0.26%; p < 0.001) for perifoveal VD and − 0.001 mm^2^ (95% CI − 0.001 to 0.002; p = 0.001) for the non-flow area for each unit increase in SSI for the comparison of images with different SSI values.

**Conclusions:**

The influence of image quality on OCTA metrics should be considered for image comparisons during follow-up to avoid misinterpretation of small changes in OCTA parameters in patients with diabetes.

## Introduction

Diabetic retinopathy (DR) is one of the most common complications of diabetes that develops in approximately 75% of diabetic patients after 10 years of disease duration [1–3]. Presence of vision-threatening complications such as retinal neovascularization and diabetic macular edema (DME) has a significant influence on quality of life [4–6]. Identification of diabetic patients with an increased risk of these complications is indispensable for the prevention of visual impairment. In the delineation of retinal vascular perfusion and macular edema, ocular imaging by fluorescein angiography and optical coherence tomography (OCT) has a significant role. Optical coherence tomography angiography (OCTA) is a new non-invasive imaging technique that is capable of visualize the blood flow in various layers of the retina and choroid without the use of intravenous dye [7]. Besides information of the retinal structure, OCTA provides a detailed view of the retinal vasculature that allows accurate visualisation of microvascular abnormalities and capillary dropout areas in retinal vascular diseases. Since the appearance of OCTA, several studies have delineated the alteration of retinal microvasculature of diabetic patients such as vascular remodeling, enlargement of the foveal avascular zone (FAZ), visualisation of microaneurysms and capillary dropout areas [8–17]. Numerous studies have described the high accuracy and reproducibility of OCTA parameters in normal subjects [18–25] as well as in patients with diabetes [26], however the influence of scan quality on the accuracy of OCTA measurements has not been examined in detail. It is well known that OCT scan quality varies greatly, depending on a number of factors, including media opacities, ocular saccades, blink artifacts, and OCT operator skills [27, 28]. The signal intensity score, as an indicator of scan quality is calculated directly from the intensity of the image acquired by the device with different ranges and recommended thresholds provided by the manufacturer. Currently the most widely available AngioVue System provides signal strength index (SSI) in order to define the image quality. The SSI is based on the intensity or brightness of the reflected light during scanning and this index ranges from 0 to 100 and is different for the distinct scan types since the anatomical features differ in their reflectivity. It has already been shown that images with higher signal intensities are associated with more accurate segmentation of retinal layers, and thus, improved repeatability of the thickness measurements [29, 30]. Various artifacts appear at different frequencies in OCTA images [31]. In previous studies, media opacities were confirmed to be a reason for signal loss during OCTA [32], and lower image quality was associated with an increase in artifact frequency and with lower measurement repeatability in healthy volunteers [33, 34]. One previous study demonstrated that cataracts can significantly influence quantitative vasculature measurements, even in high-quality images using swept-source OCTA [35]. Another recent study noted that posterior subcapsular cataract can induce a reduction in peripapillary vessel density that may falsely suggest glaucoma progression [36]. Although the effect of media opacities on OCT scan quality has already been described, the relationship between signal intensity and quantitative OCTA parameters in diabetic patients has not been evaluated, nevertheless, it has clinical consequences such as avoiding the misinterpretation of a change in OCTA parameter due to a change in signal intensity.

The purpose of this study was to evaluate the relationship between image quality and quantitative OCTA parameters in patients with diabetic retinopathy to facilitate the interpretation of OCTA results.

## Methods

**Fig. 1:**
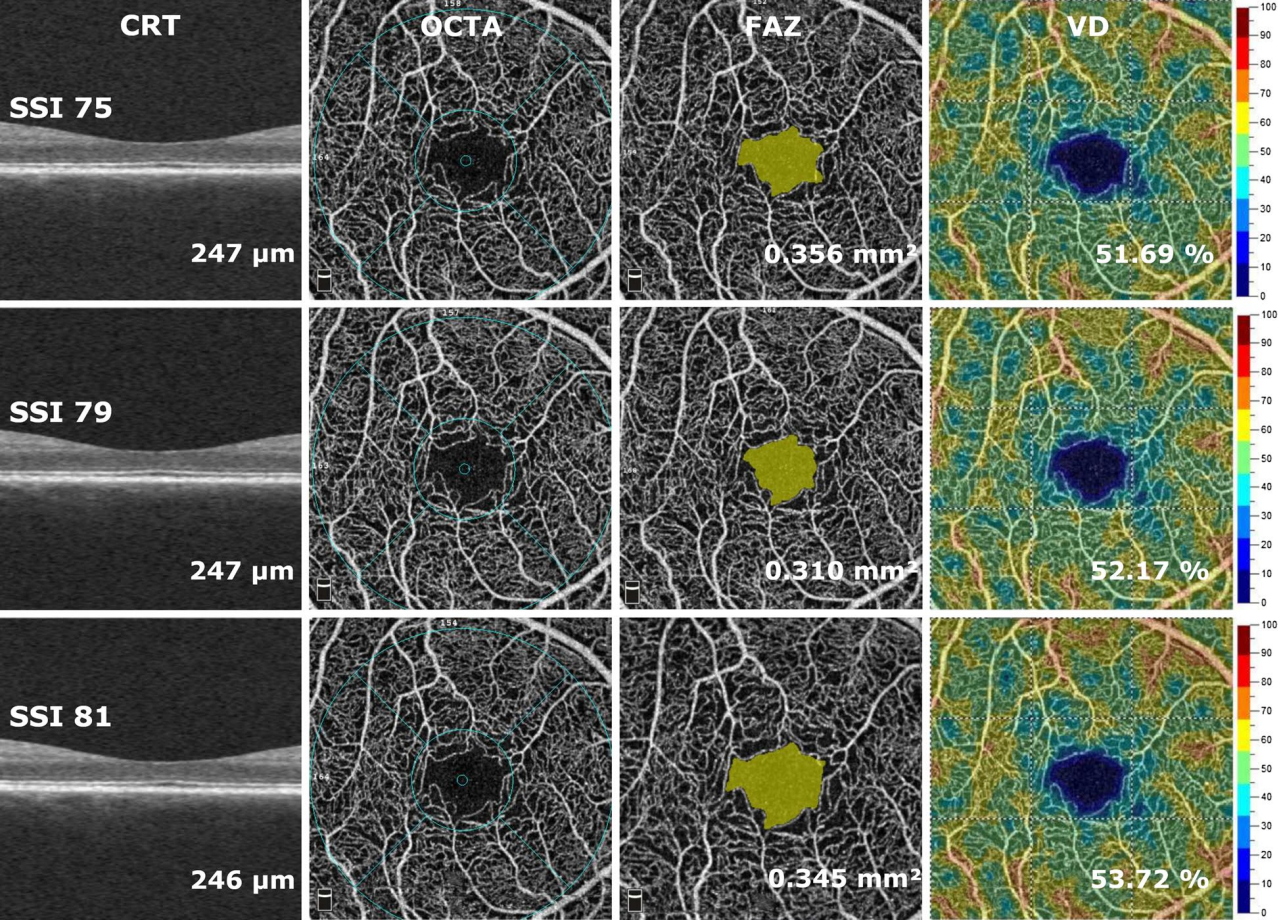
Cross sectional OCT images and *en face* OCTA imaging of the superficial retinal capillary plexus (SCP) using the automated AngioAnalytics software in a diabetic subject after the acquisition of three consecutive images from the same subject. Foveal avascular zone area and vessel density of the SCP were measured using the non-flow detection and flow density tool show substantial fluctuation across the images

A total of 100 eyes of 50 subjects with type 1 (n = 8) and type 2 (n = 42) diabetes were recruited in this prospective, observational cross-sectional study from the outpatient clinic of the Department of Ophthalmology at Semmelweis University. The study was conducted according to the Declaration of Helsinki, relevant national and local requirements, and was approved by the Ethical Review Board for Human Research of the National Drug Agency. All patients signed their written informed consent. Thirty-seven eyes of diabetic patients without any retinopathy and 63 eyes with early or moderate retinopathy, as defined by the International Clinical Diabetic Retinopathy Disease Severity Scale of the American Academy of Ophthalmology, were enrolled in the study. Exclusion criteria for the participants were any history of intraocular surgery or other ocular disease (such as age-related macular degeneration, glaucoma, vitreomacular disease), previous intraocular anti-VEGF (vascular endothelial growth factor), steroid or laser treatment, the presence of clinically significant lens opacities, or refractive error > 6 diopters. All subjects underwent a comprehensive ophthalmic examination including slit lamp and fundus examinations using indirect ophthalmoscope. Optical coherence tomographic angiography imaging was performed using the AngioVue OCTA system with an SSADA (split-spectrum amplitude decorrelation angiography) software algorithm. Retinal thickness was measured in the central area of the macula with a diameter of 1.0 mm. The AngioAnalytics software of OptoVue system with an automated segmentation algorithm was used to assess superficial vessel density (VD) in the central macular 3 × 3 mm and in the perifoveal region, as well as foveal avascular zone (FAZ) area in square millimeters (mm^2^) (Fig. 1). In this study we analyzed retinal vessel density in the superficial capillary plexus, as quantitative analysis of the deep capillary plexus is less reliable due to projection artifacts. In addition, scans with pronounced diabetic macular edema, and a consequence presence of segmentation errors at the level of the superficial vascular plexus, were excluded from the study. As a result, no segmentation line required manual modification during image analysis. According to the usual recommendation for OCTA imaging, we accepted only scans with good image quality (signal strength index (SSI) > 35) after meticulous check for the presence of autosegmentation alignment errors at the level of the superficial capillary plexus and motion artifacts due to saccadic eye motion, blinking or double vessel pattern. Each study subject underwent one session of imaging, during which three OCTA images were obtained consecutively.

### Statistical analysis

Statistical analysis was performed with Statistica software (version 13.2, Statsoft Inc., Tulsa, OK, USA). A priori sample size calculation (power = 0.90; p = 0.05, maximum allowed difference in VD: 5%) was performed as described elsewhere, [37] and provided the minimum number of eyes to be 37 eyes.

The repeatability of the OCTA parameters was characterized by the corresponding coefficient of variation (CV) values. To determine how repeatability of the OCTA parameters was related to the quality of acquired scans, both the average, and the coefficient of variation of the three consecutive OCTA measurements were plotted as a function of the mean SSI. The Cronbach’s alpha value and intraclass correlation coefficient (ICC) calculated using one-way random model were used to estimate the reliability of the consecutive measurements. Next, an analysis was made to determine how measured OCTA parameter would change with a one-unit change in SSI value. For this purpose, the effect of SSI value on OCTA parameters was assessed after controlling for the effect of the presence of DME using multivariable regression on repeated measures via generalized estimating equation (GEE) models. In GEE models, measurement data obtained consecutively from the two eyes of the same subject were statistically analyzed as repeated measures. Thus, this analysis takes into account the correlated nature of data from the two eyes of the same patient and provides valid p values for mean changes in OCTA parameters as a result of a one-unit change in SSI. In all statistical analyses, a p-value of less than 0.05 was considered to be statistically significant.

## Results

A total of 100 eyes of 50 patients (26 male and 24 female, mean age: 58.3 ± 12.6 years) consisting of 34 eyes with DME and 66 eyes without DME were included in the study. A fluctuation in OCTA metrics due to signal loss can be observed on images taken successively from the same subject as an indicator of intrasession variability of measurements (Fig. 1). Values of SSI ranged from 30 to 85, the overall mean SSI was (61.79 ± 10.38) and eyes with DME had significantly lower SSI values compared to eyes without DME (57.64 ± 11.47 vs. 63.94 ± 9.15; p = 0.003). However, the presence of DME did not show any influence on SSI variability (eyes with DME: 3.00 ± 1.77 vs. eyes without DME: 3.19 ± 2.19; p > 0.05).

**Fig. 2:**
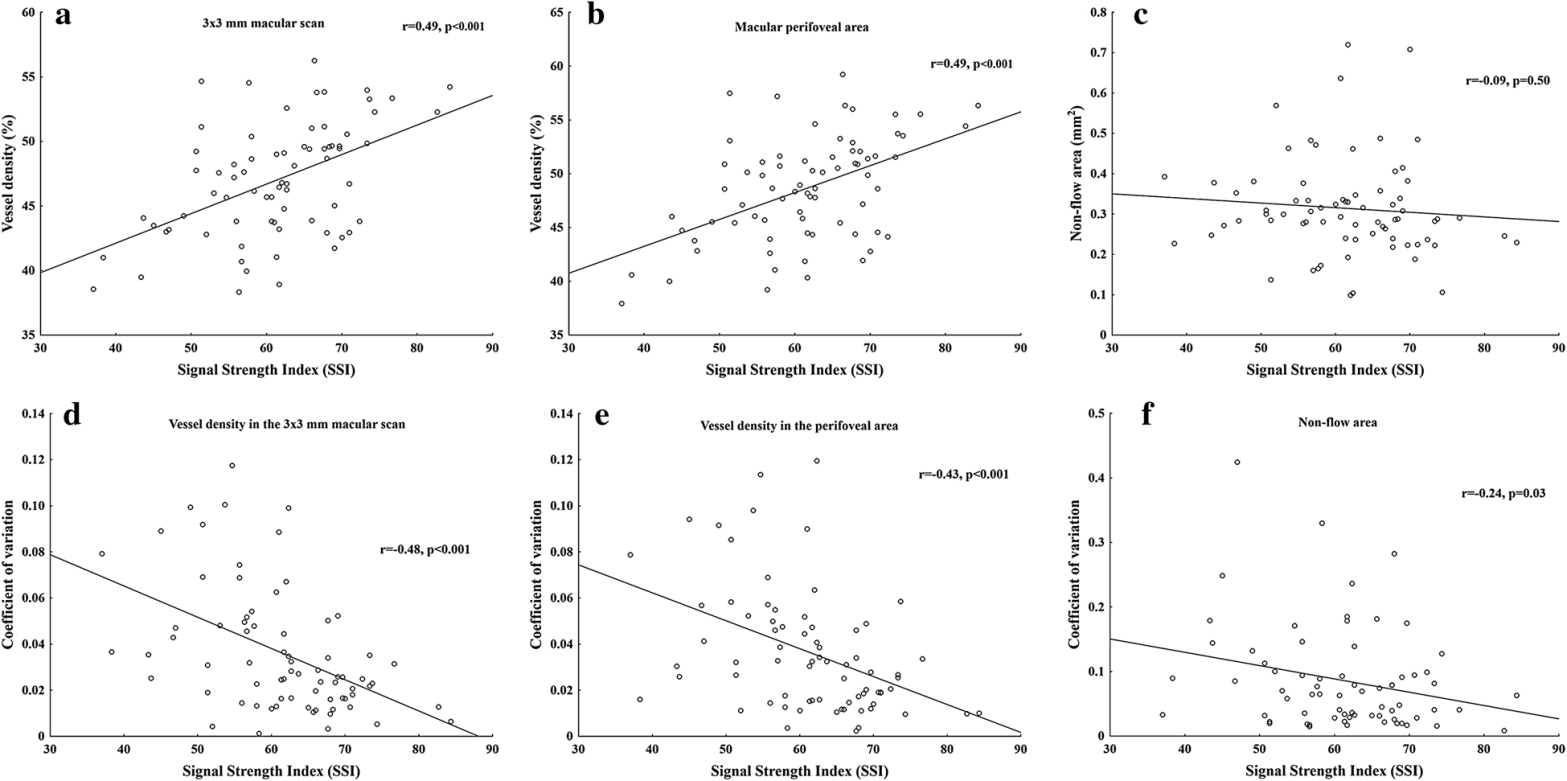
Effect of signal strength index (SSI) values on average retinal capillary vessel density measurements (**a**, **b**) and on non-flow area (**c**). Measurement error of OCTA parameters (**d**–**f**) for scans with SSI scores ranging from 30 to 85; as SSI increases, measurement repeatability improves

Mean SSI values from the three consecutive measurements showed significant positive correlation with the mean retinal capillary vessel density values (Fig. 2a, b), but not with non-flow area (Fig. 2c).

Given the assumed effect of image quality on the repeatability of measurements, we calculated regression coefficients between SSI and the corresponding coefficient of variation values for different OCTA parameters. By analysing linear relationship, we found significant negative correlation between SSI and coefficient of variation of the three consecutive measurements for vessel density (Fig. 2d, e) and for non-flow area (Fig. 2f). However, coefficient of variation values were significantly lower for vessel density measurements (3 × 3 mm scan: 3.62 ± 2.72, perifoveal scan: 3.67 ± 2.70) compared to non-flow measurements (10.21 ± 11.29, p < 0.001). Intra-rater reliability of the consecutive measurements was found to be excellent (Cronbach’s alpha: 0.96; ICC: 0.96).

In order to facilitate comparisons of scans acquired with different image quality we determined a correction factor for changes in SSI providing a mean correction factor of 0.22% (95% CI 0.20–0.24 µm; p < 0.001) for VD at the 3 × 3 mm macular scan, 0.23% (95% CI 0.21–0.26%; p < 0.001) for perifoveal VD and − 0.001 mm^2^ (95% CI − 0.001 to 0.002; p = 0.001) for the non-flow area for each unit increase in SSI after controlling for the effect of DME in multivariable models. In these multivariable analyses, the presence of DME had a significant effect on VD at the 3 × 3 mm macular scan (− 1.63%, 95% CI − 1.32 to − 1.95%, p = 0.000), on perifoveal VD (− 2.07%, 95% CI − 1.88 to − 2.27%, p = 0.000) and on non-flow (+ 0.017 mm^2^ 95% CI 0.006–0.027 mm^2^, p = 0.002) measurements independent of image quality.

## Discussion

The purpose of this study was to examine the influence of image quality on the repeatability of AngioFlow parameters in patients with diabetes using the AngioVue OCTA imaging system. Findings of the current study support that measurement error is substantially larger in scans with lower image quality compared to those with better quality. Moreover, we found that measurement repeatability is substantially better for vessel density measurements than for non-flow measurements. The importance of this finding is that with the intention of introducing this new imaging technique in routine clinical care, it is essential to define the effect of image quality on the repeatability of measurements to prevent an unnecessary increase in the number of unqualified scans, and thus, measurement time.

In this study, we analyzed the correlation between SSI and measured values of OCTA parameters to evaluate whether image quality significantly influences OCTA measurements. Since the true values of OCTA parameters should be constant during consecutive imaging the same eye in such a short time, this correlation measure only the effect of SSI on OCTA metrics. Confirming previous results, the reliability of the consecutive measurements was found to be excellent in this study [38]. We provided correction factors for each unit increase in SSI for vessel density and non-flow measurements suggesting that the artifactual bias of the SSADA algorithm of the AngioAnalytics software should be taken into consideration when interpreting OCTA results. A correction factor of 0.22% means that a decrease of 2.2% in vessel density could be measured solely owing to loss of signal intensity (SSI) by 10 on a follow-up scan resulting in the erroneous conclusion of a clinically significant change. According to the significant SSI-VD correlation even in high quality images, compensation of SSI for DR progression analysis is suggested for accurate comparisons of scans during follow-up of these patients. Our primary purpose in this study was to evaluate the effect of image quality on the reliability of OCTA in detecting retinal microvascular damage in order to make both screening and evaluation of progression more accurate.

OCTA technology allows us to investigate the retinal microcirculation [39] with high reproducibility and reliability [40] even before clinical vascular alterations can be observed on the fundus examination. In contrast to conventional OCT devices, OCTA uses flow signal instead of reflectance signal. Different OCT-based angiography algorithms including Doppler shift, speckle variance, phase variance, optical micro-angiography (OMAG), split-spectrum amplitude decorrelation angiography (SSADA) and correlation mapping are employed to contrast retinal blood vessels from static tissue by evaluating the change in the OCT-signal caused by the moving blood cells. The signal-to-noise ratio is a mathematical relationship that may influence the image quality. Low signal strength reduces the signal-to-noise ratio by decreasing the OCT signal that leads to a consequent increase in image noise. The most widely available OCTA algorithm is SSADA that significantly improved the signal-to-noise ratio of flow detection allowing better visualization of the retinal vasculature [34, 41–43].

In routine clinical setting, decreased image quality can be attributed to lens opacities or other reasons such as abrupt changes in tear film quality, moving vitreous floaters, patient cooperation or operator skills. When these causes result in the presence of artifacts such as segmentation errors, motion artifacts and shadowing artifacts on the acquired scans, these images are usually excluded from quantitative OCTA assessment of retinal microcirculation, and retaken, however, scans without visible artifacts are often accepted, despite their lower quality when image quality is above the recommended threshold. Nevertheless, our study showed that independent of the reason for decreased image quality, scan quality has a significant impact on quantitative OCTA parameters, and this has to be taken into consideration when interpreting OCTA results.

The AngioAnalytics flow density map software of the AngioVue system estimates the percentage of the total evaluated area occupied by flowing retinal blood vessels in the superficial retinal vascular layer with high accuracy [44, 45]. Although measurement error of VD and FAZ area was not affected by the presence of diabetic macular edema in high quality scans, we found significantly better image quality in eyes without intraretinal fluid compared to those with macular edema. Cystoid macular edema in diabetes involves fluid accumulation in different retinal layers, mainly in the outer plexiform layer [46]. It can reduce the contrast between different layers and as a consequence, the segmentation remains less reliable for automated methods [47]. Since we evaluated changes in the superficial capillary plexus, and scans with misidentification of segmentation lines on due to macular edema were excluded, the possible effects of segmentation error on our results could be ruled out in this study. However, we found a significant effect of DME on values of OCTA parameters independent of image quality suggesting that the presence of macular edema has to be taken into consideration when comparing OCTA parameters from diabetic patients.

The knowledge of the measurement error of a device is essential both for early detection of retinal abnormalities and for the follow-up of these patients [48–50]. Previous reports have already assessed the most important reasons why OCT measurement values may be affected by SSI values. In short, OCT software algorithms depict the inner and outer boundaries of the structure being determined by their reflectance properties [29]. Low overall illumination of the acquired image due to media opacities, floaters, blink artifacts or eye saccades can lead to less accurate segmentation and increased measurement variability. Another potential source is operator error during image acquisition, as the directional reflectance of the superficial retinal layer affects SSI, and subsequently retinal thickness and vessel density measurements. Previous studies showed that higher signal intensity scores results in thicker measurement of retinal layers using both time-domain and Fourier-domain OCT devices [51–56].

Concerning limitations of this study, the acquired data is obtained from a single center population, which may limit the generalizability of our results. However, we presume that given the number of eyes included in this study the conclusions derived from our results are reliable. Since the operator frequently must accept less than ideal scans in real world practice, further studies are needed to investigate whether the repeatability of OCTA measurements could be improved by using the latest software versions with improved scan acquisition and image analysis. Finally, whereas higher SSI values are associated with enhanced repeatability, it remains to be examined whether an SSI correction model would helps clinicians to follow DR progression. Nevertheless, any advancement that improves the ability to detect true changes in retinal microvasculature over time is appreciated, and future studies are required to assess the role of SSI on OCTA metrics in the clinical setting.

## Conclusion

The influence of image quality on measurement error should be considered during the follow-up of diabetic patients with OCTA metrics. For the RTVue OCT instrument, we suggest a correction factor of 0.22% for each unit change in SSI for vessel density for image comparisons during follow-up.

## Data Availability

All the data supporting our findings is contained within the manuscript.

## Abbreviations

FAZ: foveal avascular zone
OCTA: optical coherence tomography angiography
SSI: signal strength index
VD: vessel density
VEGF: vascular endothelial growth factor
